# Fatal myocardial fibrosis in an aged chimpanzee (*Pan troglodytes*)

**DOI:** 10.3402/pba.v3i0.21073

**Published:** 2013-06-10

**Authors:** Audrey Baldessari, Jessica Snyder, Joel Ahrens, Robert Murnane

**Affiliations:** 1 Washington National Primate Research Center, University of Washington, Seattle, WA, USA; 2 Department of Comparative Medicine, University of Washington, Seattle, WA, USA

**Keywords:** interstitial myocardial fibrosis, great apes, sudden death

## Abstract

A 36-year-old male chimpanzee (*Pan troglodytes*) assigned to a life-long sign language communication project presented for sudden death. No other clinical or clinical pathological abnormalities were noted and given the signalment, death due to cardiac failure was suspected. Necropsy findings revealed moderate cardiomegaly and other chronic age-related findings including focal renal tubular cystic dilation and gingival hyperplasia. Histologic evaluation of the heart revealed interstitial fibrosing cardiomyopathy characterized by severe interstitial myocardial fibrosis replacing and separating myofibers within all chambers of the heart, especially the left ventricle, interventricular septum and subvalvular areas. This case report represents an additional case of sudden death associated with interstitial myocardial fibrosis in a chimpanzee. This process has been previously cited as the most common cause of sudden death in aged chimpanzees.

Sudden death associated with heart failure is described as the most common cause of death in captive populations of chimpanzees and has been described in other great apes including gorillas and orangutans (1–4). The most common pathologic lesion is interstitial fibrosing cardiomyopathy, and sudden death is thought to be associated with defective conduction or sudden cardiac arrhythmias.

Several mechanisms may contribute to age-related cardiac fibrosis, including recruitment and activation of resident fibroblasts and fibroblast precursors influenced by various molecular signaling pathways such as the endothelial-mesenchymal transition mediated by TGF-β or bone marrow-derived circulating stem cells and monocytes (5, 6), and impaired reparative responses including reduced collagen degradation in the remodeling process and altered collagen cross-linking. Cardiomyocytes are inherently rich in mitochondria, which generate reactive oxygen species as a by-product of oxidative phosphorylation, influencing cytokine signaling and fibroblast metabolism (7). The mechanisms of myocardial fibrosis likely differ in the diseased, ischemic or pressure-overloaded heart as compared to the heart of a normal, aging individual.

Due to the genetic similarities, cardiac disease in aging humans has been compared to heart failure in great apes, especially the chimpanzee. The differences in presentation and lesion characteristics between non-human primates and human cardiac disease are striking in that aging chimpanzees develop a network of interstitial collagen without apparent vascular disease or myofiber injury, whereas humans typically acquire vascular occlusive disease over the course of their lives which leads to ischemic injury to myocardial fibers (8). Other non-human primate species in research settings develop varying degrees and severity of cardiac disease if fed an atherogenic diet, but with the exception of some New World primate species, rarely develop atherosclerotic changes when fed a diet similar to their foraging counterparts (9).

## Case report

A 36-year-old male chimpanzee (*Pan troglodytes*) presented to the Washington National Primate Research Center for postmortem examination following sudden and unexpected death. For the past 31 years, the animal had been housed in an indoor/outdoor facility at the Central Washington University Chimpanzee and Human Communication Institute. The animal was maintained in accordance with the Animal Welfare Act and the *Guide for the Care and Use of Laboratory Animals* (10). This chimpanzee was part of a sign language and communication research group and was closely monitored on a daily basis. There was history of a single episode of vomiting the prior week. No other premonitory signs were noted by either the research staff or clinical veterinarian. The male partner of the deceased animal inflicted postmortem integumentary wounds to the face, ventral thorax and scrotum. Differential diagnoses at presentation for postmortem examination included cardiac disease or a vascular event, such as an aneurysm, pulmonary thromboembolism, or stroke.

## Pathology

**Fig. 1:**
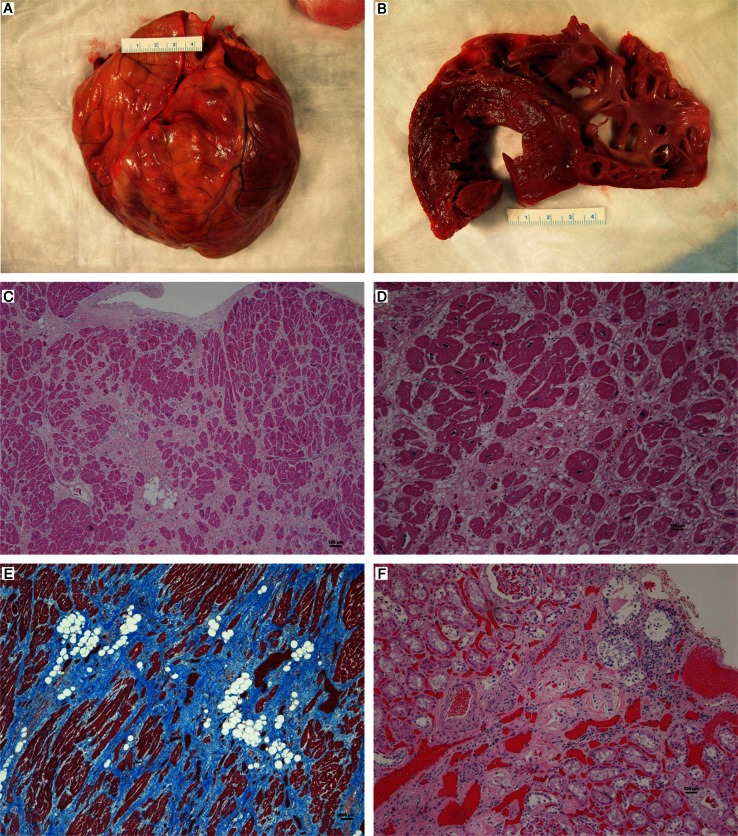
(A). Chimpanzee heart. Note the diffuse cardiomegaly, (B) cut section of the myocardium. Note the thick streaks of pallor within the papillary muscles, (C) interstitial myocardial fibrosis, with pale staining collagen separating myofibers, 40×, H&E, (D) interstitial myocardial fibrosis with adipose tissue infiltrates and mild chronic inflammation, 100×, H&E, (E) interstitial myocardial fibrosis; note the abundant blue staining collagen bands, 40×, trichrome stain, (F) chronic renal infarct with interstitial nephritis and fibrosis, 100×, H&E.

At necropsy, body weight appeared normal and was approximately 160 lbs (72.7 kg). There was moderate postmortem autolysis. Externally, there was marked, multifocal gingival hyperplasia and dorsal displacement of the right maxillary canine tooth. Multifocal, postmortem skin abrasions and lacerations were present ventrally, focused on the perioral and scrotal areas. The heart was diffusely, moderately enlarged (Fig. 1A), with a weight of 497.3 g and displacement of 217 cm^3^. There were multifocal, firm pale streaks (scarring) predominantly within the left ventricular free wall, interventricular septum, and apex of the heart, with similar though less prominent lesions seen in all chambers (Fig. 1B). The right ventricle was mildly dilated. Other lesions included a focal, discrete 2 cm diameter, multicystic lesion in the right renal cortex. Minor to moderate multifocal fibrous adhesions were noted between the loops of the small intestine and between the small and large intestine.

Tissues were fixed in 10% neutral buffered formalin, processed routinely, embedded in paraffin and stained with hematoxylin and eosin (H&E). Histologic examination of the heart revealed multifocal, moderate to severe, mostly mature fibrosis separating and replacing myofibers in all chambers (Fig. 1C and D). Islands of mature adipose tissue were also present in the fibrotic areas, especially within the atria. There was significant fibrosis at the base of the mitral valve. Trichrome staining revealed abundant blue stained collagen separating remaining myofibers (Fig. 1E). There were small numbers of lymphocytes and histiocytes diffusely spread within the areas of fibrosis or surrounding degenerate myofibers and within the epicardial connective tissue. Multifocal, moderate to marked anisokaryosis was seen within the remaining myofibers. Minimal, light brown, perinuclear granular pigment (lipofuscin) was seen in scattered myocytes. Rare, minimal to mild atherosclerotic changes were seen in random coronary arterioles, characterized by few mural macrophages, foamy cells and eosinophilic matrix material. Other age-related findings included a focal nodule of renal cortical tubular ectasia and few renal cortical infarcts with fibrosis and chronic inflammation (Fig. 1F). Brain was not available for examination.

## Discussion

Sudden cardiac death has been previously described in great apes and the prevalence of sudden death associated with heart disease in chimpanzees has been reported to be as high as 68% in captive adult populations, with males more often affected than females (3, 8, 11–13). This case represents an aged adult male chimpanzee that died unexpectedly of heart disease. With the exception of a single episode of vomiting, no clinical signs were seen prior to death and clinical pathology data were not available. The heart weight-to-body weight ratio (0.0068) observed postmortem was consistent with moderate cardiomegaly, with a previously reported mean heart weight-to-body weight ratio in chimpanzees with grossly and microscopically normal hearts of 0.0052 (1). Significant remodeling of the interstitium was seen microscopically. In the largest case report describing a group of 36 animals, 36% of which died of sudden cardiac death, the average age of animals with moderate cardiac fibrosis was 28.9 years with a range 21–40 years (1). This disease is typically reported retrospectively as controlled studies of cardiac disease are not possible in this species. Death in these cases is speculated to be due to conduction abnormalities.

The predominant histologic lesion in this animal was moderate to severe interstitial cardiac fibrosis. Similar to other case reports of sudden cardiac death with fibrosis (13), the coronary arteries lacked evidence of significant atherosclerotic changes. Atherosclerosis in humans is considered to be an inflammatory disorder with chronic inflammation of coronary arteries occurring secondary to metabolic changes such as an atherogenic lipid profile (elevated low-density lipoprotein [LDL] and cholesterol, and decreased high-density lipoprotein [HDL] associated with aging). Such arterial changes lead to sudden coronary arterial occlusion or chronic partial occlusion with ischemia, remodeling, and fibrosis via repair mechanisms. There have been comparisons between interstitial myocardial fibrosis in chimpanzees and other great apes, and humans, although the latter tend to develop scarring and fibrosis secondary to vascular disease with infarction and ischemia (1). Despite the close genetic relationships between humans and great apes, and the similar progressive weight gain and atherogenic lipid profile with age in male chimpanzees (14), the etiology of cardiac fibrosis in apes does not appear to be vascular in origin. The amount of interstitial inflammation observed in non-human primate cases varies within the literature but was mild in this case. An infectious process such as *Trypanosoma cruzi* or viral disease was considered unlikely because no histological evidence was present and the chimpanzee did not have a compatible travel history. Amyloidosis has been described concurrently with cardiac fibrosis in one chimpanzee and was thought to be associated with chronic inflammation although no histologic examination was described in that retrospective report (2). Often no significant inflammation or atherosclerosis is seen in these cases (1, 3, 8).

Dietary differences may play a role in the lack of development of atherosclerotic lesions, as the majority of non-human primates in captivity consume a commercial primate diet, which is typically low fat (approximately 5–10%) and supplemented with fruits and vegetables. The average LDL of Americans is 130 mg/dl, while free ranging baboons on a foraging diet have reported values of 40–80 mg/dl (15). However, adult captive non-human primates tend to weigh more than their corresponding free ranging species and in captivity, like humans, tend to become obese with age. Lipid profiles of both groups also tend to change similarly with age (14, 16, 17). It is reported that while other primate species, notably rhesus macaques (*Macaca mulatta*), do not develop atherosclerosis naturally, they do form atherosclerotic plaques if fed an atherogenic diet resulting in hypercholesterolemia (9, 18). Cynomolgous macaques (*Macaca fascicularis)* have been shown to display a marked difference in development of atherosclerotic lesions related to age, with young animals (2.5–3.5 years of age) showing less coronary artery lesions despite similar serum lipid concentrations as compared to adults (6–12 years of age) (19, 20). Other metabolic factors that tend to change with age such as central obesity (waist circumference), hyperinsulinemia, accumulation of glycation end products, and increased blood pressure, all play a role in human coronary vascular disease. Glucose tolerance decreases with age, and glycation end products have been shown to accumulate in arteries even in non-diabetic cynomolgous monkeys (9, 19) but this has not been studied in chimpanzees.

Early diagnosis of heart disease may assist in clinical management. In human medicine, investigation into the potential uses of biomarkers of inflammation, oxidative stress, myocyte injury, and collagen metabolism has been investigated (21). In one report, brain-type natriuretic protein, a peptide which correlates positively with increased cardiac wall tension and heart enlargement, and cardiac troponin I, which is released due to myocardial necrosis, were both elevated in 28 cases of various types of cardiovascular disease in chimpanzees (17). C-reactive protein and a lipid panel, including cholesterol, LDL and triglycerides, were not useful clinically in the diagnosis of heart disease in chimpanzees, although the lipids notably increased with age (17). Biomarkers of collagen metabolism have been investigated for potential use in the clinical management of great apes. A human assay for matrix metalloproteinase-1 did not cross-react with chimpanzee sera but initial carboxy terminal telopeptide and pro-collagen III n-terminal protein did increase in cases of cardiovascular disease that had concurrent renal disease (22). Approximately two thirds of the cells in the myocardium are fibroblasts, which compose a variable proportion of the heart volume and influence cell signaling between cardiomyocytes via the extracellular matrix and molecular chemical mediators (23, 24). In health, type I collagen predominates (approximately 80%) and type III collagen comprises approximately 20%, with lesser amounts of type IV collagen. Alterations in the proportions of collagen may lead to changes in cardiac elasticity (25); however, no data are found specifically on changes in collagen types in cases of cardiac fibrosis in great apes. Collagen breakdown products are not specific to cardiac fibroblasts and heart disease, and may also increase in association with collagen turnover at other sites (26), and rates of collagen breakdown may not correlate with collagen deposition in the myocardial interstitial space. Due to the clinically silent nature of fibrosing cardiomyopathy in great apes, a screening test or panel that is reactive with primate sera, and both specific and sensitive would be ideal, however, more work is needed in this area. Other measures such as blood pressure and functional parameters including heart rate, cardiac output, and mural compliance may be helpful to characterize cardiac function in aging chimpanzee populations with a goal of detecting developing heart disease at earlier stages.
